# Male Infertility: Diagnostic Approach – A Committee Opinion

**DOI:** 10.1590/S1677-5538.IBJU.2025.0223

**Published:** 2025-05-05

**Authors:** Sandro C. Esteves, Marina C. Viana, Augusto B. Reis, Filipe Tenório Lira, Thiago Afonso Teixeira, João Paulo Camarço, Matheus Gröner, Antônio José T. Paula, Alberto C. Stein, Maria Gabriela F. Mulato, Jorge Hallak, Renato Fraietta

**Affiliations:** 1 ANDROFERT Clínica de Andrologia e Reprodução Humana Campinas SP Brasil ANDROFERT, Clínica de Andrologia e Reprodução Humana, Campinas, SP, Brasil; 2 Universidade Estadual de Campinas Faculdade de Ciências Médicas Departamento de Cirurgia Campinas SP Brasil Departamento de Cirurgia (Disciplina de Urologia), Faculdade de Ciências Médicas, Universidade Estadual de Campinas - UNICAMP, Campinas, SP, Brasil; 3 Faculdade de Medicina da Universidade Federal de Minas Gerais Departamento de Cirurgia e Programa de Pós-graduação em Ciências Aplicadas à Cirurgia e Oftalmologia Belo Horizonte MG Brasil Departamento de Cirurgia e Programa de Pós-graduação em Ciências Aplicadas à Cirurgia e Oftalmologia, Faculdade de Medicina da Universidade Federal de Minas Gerais - UFMG, Belo Horizonte, MG, Brasil; 4 Laboratório de Reprodução Prof. Aroldo Fernando Camargos Serviço de Urologia Belo Horizonte MG Brasil Serviço de Urologia, Laboratório de Reprodução Prof. Aroldo Fernando Camargos, Belo Horizonte, MG, Brasil; 5 Universidade Federal de Pernambuco Departamento de Cirurgia Recife PE Brasil Departamento de Cirurgia, Universidade Federal de Pernambuco - UFPE, Recife, PE, Brasil; 6 Instituto de Medicina Integral Prof. Fernando Figueira & Clínica Andros Recife Recife PE Brasil Instituto de Medicina Integral Prof. Fernando Figueira & Clínica Andros Recife, Recife, PE, Brasil; 7 Universidade Federal do Amapá Faculdade de Medicina Departamento de Cirurgia Macapá AP Brasil Disciplina de Urologia, Departamento de Cirurgia, Faculdade de Medicina, Universidade Federal do Amapá - UNIFAP, Macapá, AP, Brasil; 8 Universidade de São Paulo Instituto de Estudos Avançados Grupo de Estudos em Saúde Masculina São Paulo SP Brasil Grupo de Estudos em Saúde Masculina, Instituto de Estudos Avançados, Universidade de São Paulo - IEA-USP, São Paulo, SP, Brasil; 9 ANDROSCIENCE Centro de Ciência e Inovação em Andrologia & Laboratório Clínico e de Pesquisa de Alta Complexidade São Paulo SP Brasil ANDROSCIENCE, Centro de Ciência e Inovação em Andrologia & Laboratório Clínico e de Pesquisa de Alta Complexidade, São Paulo, SP, Brasil; 10 Hospital Estadual Alberto Rassi Departamento de Urologia Goiânia GO Brasil Departamento de Urologia, Hospital Estadual Alberto Rassi, Goiânia, GO, Brasil; 11 Humana Medicina Reprodutiva & Urocenter Goiânia GO Brasil Humana Medicina Reprodutiva & Urocenter, Goiânia, GO, Brasil; 12 Universidade Federal de São Paulo Departamento de Cirurgia Disciplina de Urologia São Paulo SP Brasil Disciplina de Urologia, Departamento de Cirurgia, Universidade Federal de São Paulo - UNIFESP-EPM, São Paulo, SP, Brasil; 13 Universidade Federal de São Paulo Setor Integrado de Reprodução Humana São Paulo SP Brasil Setor Integrado de Reprodução Humana, Universidade Federal de São Paulo -UNIFESP-EPM, São Paulo, SP, Brasil; 14 ANDROLIFE Centro Integrado de Saúde do Homem Rio de Janeiro RJ Brasil ANDROLIFE, Centro Integrado de Saúde do Homem, Rio de Janeiro, RJ, Brasil; 15 Vida Medicina Reprodutiva Rio de Janeiro RJ Brasil Vida Medicina Reprodutiva, Rio de Janeiro, RJ, Brasil; 16 Cellmed - Terapia Celular e Medicina Regenerativa Divisão de Oncofertilidade Porto Alegre RS Brasil Divisão de Oncofertilidade, Cellmed - Terapia Celular e Medicina Regenerativa, Porto Alegre, RS, Brasil; 17 Faculdade de Medicina da Universidade de São Paulo Departamento de Patologia, Unidade de Toxicologia Reprodutiva São Paulo SP Brasil Departamento de Patologia, Unidade de Toxicologia Reprodutiva, Faculdade de Medicina da Universidade de São Paulo - FMUSP, São Paulo, SP, Brasil; 18 Hospital das Clínicas da Faculdade de Medicina da Universidade de São Paulo Departamento de Cirurgia Disciplina de Urologia São Paulo SP Brasil Disciplina de Urologia, Departamento de Cirurgia, Hospital das Clínicas da Faculdade de Medicina da Universidade de São Paulo - FMUSP, São Paulo, SP, Brasil; 19 Instituto ANDROSCIENCE, de Ciência, Educação e Projetos Avançados em Saúde Masculina São Paulo SP Brasil Instituto ANDROSCIENCE, de Ciência, Educação e Projetos Avançados em Saúde Masculina, São Paulo, SP, Brasil

## INTRODUCTION

Male infertility is a disorder of the male reproductive system recognized as a global health issue (1, 2). Its most common causes include congenital, genetic, anatomical, endocrine, functional and immunological factors, infections of the genital tract, cancer and its treatments, and sexual dysfunctions that prevent natural intercourse (1–5). Critical contributors include poor lifestyle habits, exposure to toxic substances, environmental influences, and advanced paternal age, which may act independently or exacerbate known causal factors (1–5).

Approximately 17% of couples of reproductive age experience difficulty conceiving. In about 20% of cases, infertility is exclusively male-related, and when combined with female factors, this percentage exceeds 50% (1, 2, 6).

## WHEN SHOULD EVALUATION BE INITIATED?

Male infertility should be investigated concurrently with female assessment in couples attempting conception for 12 months or more (1–5). In women aged 35 years or older, this evaluation should begin after six months of unprotected intercourse.

**Figure 1:**
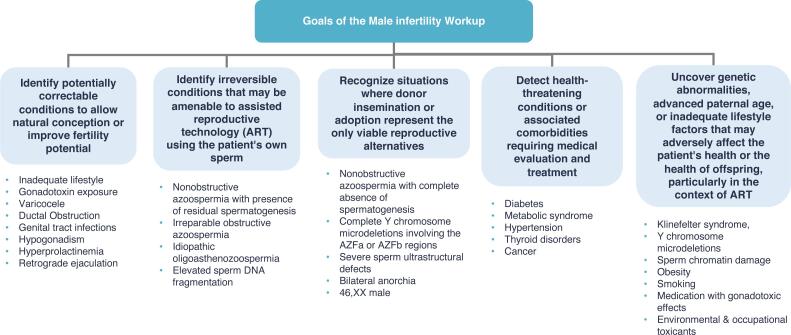
Goals of the male infertility diagnostic approach, including identifying correctable and irreversible conditions, recognizing when donor insemination or adoption are necessary, detecting health-threatening comorbidities, and uncovering genetic or lifestyle factors that may impact patient or offspring health, especially in the context of assisted reproductive technology (ART).

The goals of the evaluation are to identify (Figure-1):

- reversible causes;
- irreversible conditions amenable to assisted reproductive technology (ART) using the partner's sperm;
- irreversible conditions where heterologous gametes/embryos or adoption are the only options;
- underlying medical conditions that may impact overall male health;
- genetic abnormalities that could affect the offspring.

## HOW SHOULD EVALUATION BE CONDUCTED?

**Figure 2:**
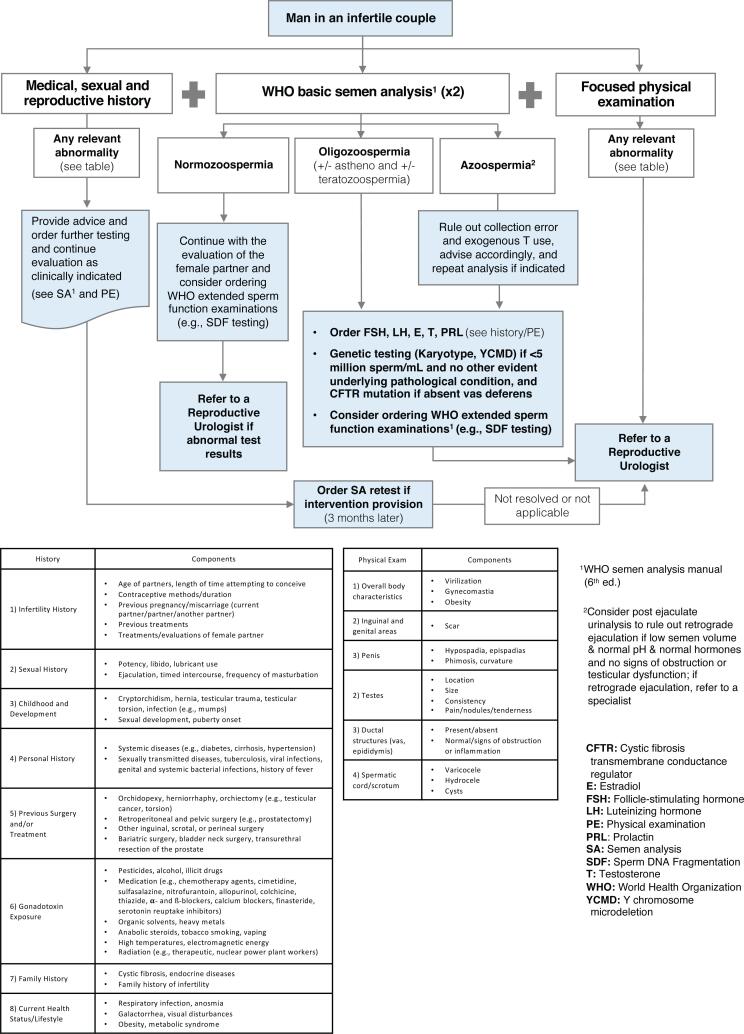
Algorithm for the initial evaluation of the male partner in an infertile couple. The process begins with a thorough medical, sexual, and reproductive history, followed by a focused physical examination. At least two semen analyses should be obtained according to WHO guidelines. Subsequent steps include laboratory testing (e.g., hormonal profile, genetic testing), imaging, and extended sperm function testing, including sperm DNA fragmentation analysis, as clinically indicated. The goal is to guide diagnosis and management tailored to the identified abnormalities.

As a disease of the male reproductive system, male infertility warrants a diagnostic work-up that goes beyond basic semen analysis. A comprehensive and standardized approach should include the following components (1–5, 7; Figure-2):

### Detailed Medical History

- Infertility history, sexual habits, childhood, and pubertal development (e.g., delayed puberty, cryptorchidism, hypospadias, epispadias, hernia, mumps orchitis, testicular trauma, or torsion).
- Prior systemic illnesses and history of sexually transmitted infections.
- Surgical history (e.g., orchidopexy, herniorrhaphy, pelvic, scrotal, or pituitary surgery).
- Exposure to gonadotoxins (e.g., pesticides, marijuana, anabolic steroids, medications including alpha/beta blockers, calcium channel blockers, anti-depressants, opioids, chemotherapy, and radiotherapy).
- Family history (infertility, endocrine disorders, cystic fibrosis, Kartagener syndrome).
- Current lifestyle (diet, alcohol intake, tobacco use, recreational and prescription drugs, physical activity, occupation).

### Physical Examination

- Evaluation of secondary sexual characteristics.
- General and focused genital exam assessing for gynecomastia, surgical scars, testicular size and consistency, epididymal and vas deferens palpation, and spermatic cord assessment.
- Clinical diagnosis of varicocele while standing in a temperature-controlled room:
  - Grade I: veins palpable with Valsalva maneuver
  - Grade II: veins palpable at rest
  - Grade III: veins visible at rest

### Semen Analysis

Conducted per the World Health Organization (WHO) 6th edition guidelines, after 2–7 days of ejaculatory abstinence (preferably 2–3 days) (8, 9) (Table-1). At least two analyses are recommended, especially when the first is abnormal. No consensus exists on the ideal interval between collections, but a two-week gap is suggested.

**Table 1:** World Health Organization reference limits for basic semen analysis parameters (6th edition, 2021).

	Centiles		
	5th	50th	90th
**Semen volume (mL)**	1.4	3.0	5.5
**Sperm concentration (x10^6^/mL)**	16	66	166
**Total sperm number (x10^6^ per ejaculate)**	39	210	561
**Total Motility (%)**	42	64	83
**Progressive motility (%)**	30	55	71
**Normal forms (%)**	4	14	32
**Vitality (%)**	54	78	95

Data were derived from approximately 3,500 men whose partners achieved a natural pregnancy resulting in a live birth within one year of unprotected intercourse. The values represent the pooled distribution of semen analysis results, with the fifth percentile considered the lower reference limit to assist clinical decision-making.

Advanced sperm function tests such as sperm DNA fragmentation (SDF) analysis should be considered in couples with recurrent pregnancy loss (natural or ART conception), unexplained male infertility, or before the use of assisted conception, including intrauterine insemination (IUI), conventional in vitro fertilization (IVF), and intracytoplasmic sperm injection (ICSI) (10).

### Hormonal Evaluation

- Indicated for men with abnormal semen parameters (especially sperm concentrations <10 million/mL), clinical signs of hypogonadism, gynecomastia, sexual dysfunction, or suspected endocrine disorders.
- The minimum hormonal panel includes serum follicle-stimulating hormone (FSH) and total testosterone. It should ideally be supplemented with luteinizing hormone (LH), estradiol, prolactin, sex hormone-binding globulin (SHBG), thyroid-stimulating hormone (TSH), free thyroxine (T4L), and calculated free testosterone.

### Genetic Evaluation

- Mandatory in cases of non-obstructive azoospermia or severe oligozoospermia (sperm concentration <5 million/mL), given that genetic abnormalities are present in up to 15% of infertile men.
- The basic genetic work-up includes G-banded karyotype analysis and Y-chromosome microdeletion testing.
- Cystic fibrosis transmembrane conductance regulator (CFTR) gene mutation testing should be performed in patients with congenital absence of the vas deferens, as mutations are present in up to 80% of such cases.

### Imaging Studies

- Scrotal ultrasound is indicated for unclear physical examination findings, palpable testicular masses, a history of cryptorchidism, and non-obstructive azoospermia due to elevated testicular cancer risk.
- Transrectal ultrasound or pelvic magnetic resonance imaging (MRI) may be required when ejaculatory duct obstruction is suspected.
- A pituitary MRI or computerized tomography scan is advised for cases with hyperprolactinemia.

### Testicular Biopsy

- Reserved for selected azoospermic patients to differentiate between obstructive and non-obstructive causes (11).
- It may be performed via percutaneous or open techniques; specimens should be preserved in Bouin or Zenker solution.
- Preferably conducted in facilities equipped for sperm cryopreservation.

## CONCLUSIONS

Infertility affects a significant proportion of men of reproductive age. Understanding the nuances of its diagnosis is essential for guiding effective treatment. A thorough and structured evaluation enables the identification of underlying causes and facilitates tailored therapeutic strategies, ultimately improving the likelihood of conception—either naturally or via ART.
